# Homogenous Cr and C Doped 3D Self-Supporting NiO Cellular Nanospheres for Hydrogen Evolution Reaction

**DOI:** 10.3390/ma15207120

**Published:** 2022-10-13

**Authors:** Zhaojun Tan, Chuanbin Li, Lijun Wang, Mingjie Kang, Wen Wang, Mingqi Tang, Gang Li, Zaiqiang Feng, Zhenwei Yan

**Affiliations:** 1 School of Mechanical Engineering, North China University of Water Resources and Electric Power, Zhengzhou 450045, China; 2 School of Materials Science and Engineering, North China University of Water Resources and Electric Power, Zhengzhou 450011, China

**Keywords:** hydrogen evolution reaction, NiO, electrocatalysts, nanosphere

## Abstract

Hydrogen evolution reaction (HER) is one promising technique to obtain high-purity hydrogen, therefore, exploiting inexpensive and high-efficiency HER electrocatalysts is a matter of cardinal significance under the background of achieving carbon neutrality. In this paper, a hydrothermal method was used to prepare the Cr-NiC_2_O_4_/NF (Ni foam) precursor. Then, the NiO-Cr-C/NF self-supporting HER catalyst was obtained by heating the precursor at 400 °C. The catalyst presents a 3D cellular nanospheres structure which was composed of 2D nanosheets. Microstructure characterization shows that Cr and C elements were successfully doped into NiO. The results of electrochemical measurements and density functional theory (DFT) calculations show that under the synergy of Cr and C, the conductivity of NiO was improved, and the Gibbs free energy of H* (∆GH*) value is optimized. As a result, in 1.0 M KOH solution the NiO-Cr-C/NF-3 (Ni:Cr = 7:3) HER catalyst exhibits an overpotential of 69 mV and a Tafel slope of 45 mV/dec when the current density is 10 mA·cm^−2^. Besides, after 20 h of chronopotentiometry, the catalytic activity is basically unchanged. It is demonstrated that C and Cr co-doping on the lattice of NiO prepared by a simple hydrothermal method and subsequent heat treatment to improve the catalytic activity and stability of the non-precious metal HER catalysts in an alkaline medium is facile and efficient.

## 1. Introduction

Hydrogen is supposed to be one of the basic energy sources that has the opportunity to replace fossil fuels in the future, due to its renewability, large-scale industrial production, high heating value, and green cleanliness. Electrocatalytic water splitting is an efficient method for producing high-purity hydrogen. Electrochemical hydrogen evolution reaction (HER) can be considered the basis of the simplest electrochemical reaction and the study of more complex multiple electron-proton transfer reactions. Despite its simplicity, it is still a multi-step chemical reaction involving three processes of adsorption, reduction and desorption on the electrode surface. Depending on the nature of the electrode, it follows different reaction pathways [1]. Platinum group metals (Pd, Ru, Rh, Ir, etc.) have always been regarded as the best HER catalysts with high activity and stability. However, their practical application has always been limited due to the high price as well as scarce reserves [2,3,4,5,6]. Researchers have been devoted to reducing or even replacing precious metals by designing efficient non-precious metal electrocatalysts to improve the reaction kinetics of HER [7,8,9].

Transition metal oxides (TMOs) are widely adopted as high-efficiency oxygen evolution reaction (OER) catalysts in alkaline electrolytes on account of their relatively low-cost and environment friendliness [10,11,12,13,14,15]. Unfortunately, high performing acidic medium-based TMOs are generally considered to be HER sluggishness due to the requirements of high overpotentials to initiate the catalysis along with the poor power efficiencies in alkaline medium. Therefore, improving the HER catalytic performance of TMOs is of great benefit to the fabrication of OER/HER dual-functional catalysts in alkaline electrocatalytic water splitting. Researchers have been committed to exploring efficient TMO electrocatalytic materials for HER used in water-splitting under alkaline conditions. In particular, nickel (Ni)-based materials, such as oxides, chalcogenides, phosphides, nitrides, alloys, etc., especially, nickel oxides have attracted much attention due to their low cost and high corrosion resistance. Qiao et al. prepared NiO nanorods (NRs) with abundant oxygen vacancies by a facile cation exchange method. Based on experimental investigations and DFT calculations, it was confirmed that the electronic structure of NiO was successfully optimized by introducing oxygen vacancies. The surface oxygen vacancies significantly enhanced the electrical conductivity of NiO NRs and also promoted the reaction kinetics of hydrogen evolution. The above-mentioned NiO NRs exhibit good alkaline HER catalytic activity and durability, outperforming standard Pt and RuO_2_ catalysts [16].

Nevertheless, the in-depth understanding of the catalytic mechanism of TMOs is still limited, and still has great potential for making further progress in transition metal oxide-based catalysts competing with the most advanced catalysts. Meanwhile, the inherently poor electronic conductivity of TMOs is another issue that hinders the further enhancement of the HER activity [17]. The use of TMOs alone as electrocatalytic materials cannot achieve the desired effect. Researchers expect that the stability and conductivity of TMOs can be improved by some means, meanwhile maintaining high electrocatalytic activity. Dai et al. combined NiO with carbon nanotubes to improve the conductivity of the catalyst; however, it is quite difficult to construct highly dispersed nano-scale oxide catalysts on conductive support conductors [18]. Suib et al. introduced O vacancies on MoO_3_ to promote its electronic conductivity and sequentially enhanced the intrinsic activity of the HER [19]. Xiaobo Chen et al. synthesized amorphous Ni@NiO nanosheets using solution growth and hydroprocessing methods [20]. The amorphous NiO shell showed good catalytic activity, which was beneficial to the Volmer and Heyrovsky step; meanwhile, the metal Ni core reduced the resistance of the catalyst, which is conducive to electron transport. Li et al. successfully doped C into NiO by electrodeposition. DFT results revealed that carbon elements may accelerate the water splitting and lower the H_2_O electrolyzation energy barrier from 1.17 eV to 0.81 eV compared with the catalyst without carbon doping [21]. Wang et al. prepared NiOx@BCNTs by calcining melamine/nickel nitrate, and the overpotential was 79 mV at 10 mA·cm^−2^ [22]. It shows that doping nickel oxide to improve its stability and conductivity is effective. One of the earliest discoveries of hydrogen evolution on Cr metal was observed accidentally, while ball-milling Cr in water [23]. Cr-based catalysts for electrochemical hydrogen evolution in alkaline media have emerged in recent research [24]. As a group VI transition metal, chromium with a half-filled d-orbital configuration may possess strong adsorption strength for the molecular species and may help improve electrocatalytic hydrogen evolution, when used as a co-catalyst [25]. Combining TMOs, especially NiO with Cr, is a feasible method to improve the HER activity, stability and conductivity of catalytic materials under alkaline conditions. In this work, the hydrothermal-annealing two-step method was applied to fabricate NiO-Cr-C/NF self-supporting HER catalyst. Through electrochemical analysis, morphology observation and DFT calculation, the effect of C and Cr co-doping on the HER activity of NiO under alkaline conditions was deeply explored and its mechanism was revealed. We hope to adopt a simple hydrothermal method and subsequent heat treatment to obtain facile and efficient C and Cr co-doped NiO non-precious metal HER catalysts, which can reduce the dependence of HER catalysts on precious metals and obtain HER electrocatalytic activity that is equivalent to that of precious metals.

## 2. Experiment

All reagents, including nickel foam (Lizhiyuan Co. Zhengzhou, China), Cr (NO_3_)_3_·9H_2_O (99.0%, Sinopharm Chemicals), Ni(NO_3_)_2_·6H_2_O (98.0%, Sinopharm Chemicals),Pt/C catalysts (10 wt%, Sinopharm Chemicals), diethyl oxalate (99.0%, Sinopharm Chemicals), KOH (95%, aladdin), ethanol (99.5%, aladdin), and Nafion (5 wt%,DuPont) were purchased from commercial suppliers and used without further purification. Milli-Q water of 18 MΩ cm was used in all experiments.

**Synthesis of NiO-Cr-C/NF-3**. The typical synthetic experiments of NiO-Cr-C/NF-3 were carried out as follows: Firstly, the nickel foams were washed with 3 M HCl solution, absolute ethanol, acetone and deionized water, respectively, under ultrasonic conditions, and vacuum dried at 60 °C for 2 h. Then 0.84 mmol Ni(NO_3_)_2_·6H_2_O and 0.36 mmol Cr (NO_3_)_3_·9H_2_O were dissolved in a solution consisting of mixed 30 mL absolute ethanol and 1.92 mL diethyl oxalate. Subsequently, we transferred the solution to a 50 mL tetrafluoroethylene reactor. After that, one piece of previously dried nickel foams was tilted against the wall of the reactor, and then the tetrafluoroethylene reactor was sealed and then placed in an oven for 12 h at 100 °C. When the reactor cooled to room temperature, the sample was ultrasonically washed for 30 s to remove excess powder on the foam. After being rinsed twice with ultrapure water and ethanol, the sample was dried in a vacuum oven at 60 °C for 2 h. Finally, the sample was annealed in an Ar atmosphere at 400 °C for 20 min with a heating rate of 5 °C/min. The catalyst was recorded as NiO-Cr-C/NF-3.

**Synthesis of NiO-C/NF, NiO-Cr-C/NF-1, NiO-Cr-C/NF-2**. Compared with the synthesis of NiO-Cr-C/NF-3, 0.84 mmol Ni(NO_3_)_2_·6H_2_O and 0.36 mmol Cr (NO_3_)_3_·9H_2_O were replaced by 1.2 mmol Ni(NO_3_)_2_·6H_2_O and 0 mmol Cr (NO_3_)_3_·9H_2_O, 1.08 mmol Ni(NO_3_)_2_·6H_2_O and 0.12 mmol Cr (NO_3_)_3_·9H_2_O, 0.96 mmol Ni(NO_3_)_2_·6H_2_O and 0.24 mmol Cr (NO_3_)_3_·9H_2_O, respectively. The other procedure is the same as that for the synthesis of NiO-Cr-C/NF-2. Four kinds of catalysts, named NiO-C/NF, NiO-Cr-C/NF-1, NiO-Cr-C/NF-2 and NiO-Cr-C/NF-3, respectively, have the mole ratio of Ni(NO_3_)_2_·6H_2_O and Cr (NO_3_)_3_·9H_2_O as 10:0, 9:1, 8:2 and 7:3. In other words, the mole ratio of Ni:Cr of NiO-C/NF, NiO-Cr-C/NF-1, NiO-Cr-C/NF-2 and NiO-Cr-C/NF-3 are 10:0, 9:1, 8:2 and 7:3.

**Preparation of 10% Pt/C/NF catalyst.** Twenty milligrams of commercial Pt/C powder (10 wt% of Pt) was dispersed in the mixture of 500 μL absolute ethanol, 500 μL deionized water and 50 μL 5% Nafion solution. Subsequently, the mixture was sonicated for 30 min to form a homogeneous solution. Lastly, the ink was dropped on the Ni foam and dried in air.

**Structural characterizations.** The powder X-ray diffraction (XRD) patterns of the electrocatalyst were recorded with the Rigaku Smart Lab X-ray diffractometer (XRD) with Cu-Kα (λ = 0.15406 nm) as the radiation source at 40 kV and 30 mA, and the scanning range 10–80° at 5°/min. Field emission scanning electron microscopy (FESEM) images were collected with the FEI Quanta 400 FEG field emission scanning electron microscope equipped with an EDS spectrometer. In the FESEM testing, a small piece of nickel foam loading the catalyst was directly adhered to the sample table with conductive glue. The transmission electron microscopy (TEM) images were obtained using an FEI Tecnai G2 F20 S-Twin field TEM at 200 kV. Before the test, the powder sample was dropped on the copper mesh. The X-ray photoelectron spectroscopy (XPS) was carried out on a Thermo ESCALAB 250 × 1 photoelectron spectrometer with an Mg Kα radiation source (λ = 1253.6 eV), and the photoelectron take-off angle was 90° with respect to the surface plane.

**Electrochemical tests.** The Standard three-electrode testing system was adopted to execute the electrochemical measurement with the CHI760E electrochemical workstation. A graphite sheet electrode, self-supporting catalyst and Hg/HgO electrode were used as the counter electrode, working electrode, and reference electrode, respectively. According to the formula, E (RHE) = E (Hg/HgO) + 0.098 + 0.0591 × pH, E (Hg/HgO) was converted to E (RHE). Linear sweep voltammograms (LSV) were recorded in the 1.0 M KOH (pH = 13.71) solution at scanning rate of 2 mV/s. The electrochemical impedance spectroscopy (EIS) was performed under 0.2 V (vs. RHE) from 0.1 to 10,000 Hz, and the amplitude of the sinusoidal voltage was 5 mV, in addition, the electrolyte was also 1.0 M KOH. The Nyquist plots were obtained based on the EIS data. The chronopotentiometry (CP) stability was applied to test the stability of the catalyst at 10 mA/cm^−2^ and 100 mA/cm^−2^ for 20 h. 

The electrochemical active surface area (ECSA) was determined by cyclic voltammetry. A series of cyclic voltammetry from 20 to 120 mV∙s^−1^ with an interval of 20 mV∙s^−1^ was collected in a non-faradaic reaction potential window from 0.2 V to 0.1 V vs. RHE. A linear plot of the *j*_anodic_ − *j*_cathodic_ versus scan rate was obtained accordingly and the slope is C_dl_ which is proportional to the ECSA. The ECSA can be calculated through the equation $ECSA=\frac{C_{dl}}{C_{s}}$. Where Cs is the specific capacitance was taken from the literature [21] (40 μF cm^−2^ for Ni-based catalysts and 30 μF cm^−2^ for Pt-based catalysts). All electrochemical measurement data were acquired without iR drop compensation.

**Computation method.** The theoretical calculation was performed using the CASTEP module of Material Studio [26,27,28]. A four-layer supercell of 2 × 2 × 2 with a vacuum layer thickness of 13 Å was used to simulate the surface of the catalyst. The generalized gradient of Perdew–Burke–Ernzerh (GGA-PBE) functionals with Hubbard U (U = 5.3 eV [29]) correction were chosen to approximate the electronic exchange related functionals [30,31,32]. The core electrons are processed by ultrasoft pseudopotentials, and plane wave cutoff energy was 490 eV. K-point mesh of 3 × 3 × 1 was used for DOS calculation and structural optimization. In addition, for all calculations, the convergence standards for energy and force are 5 × 10^−7^ eV/atom and 0.01 eV∙A^−1^, respectively. In addition, the Gibbs free energy of H* (ΔGH*) on the surface was obtained by the equation proposed by Norskov: $\Delta G_{H}=E_{surf-H}-E_{surf}-0.5E_{H2}+\Delta E_{ZPE}-T\Delta S$, where $E_{surf-H}$ was the total energy of the H adsorption surface, $E_{surf}$ was the energy of the surface, $E_{H2}$ was the energy of H2, $\Delta E_{ZPE}$ was the zero point energy, ΔS was the entropy change, and $\Delta E_{ZPE}-T\Delta S$ value of 0.134 was used as reported in the literature [21].

## 3. Result

**Figure 1:**
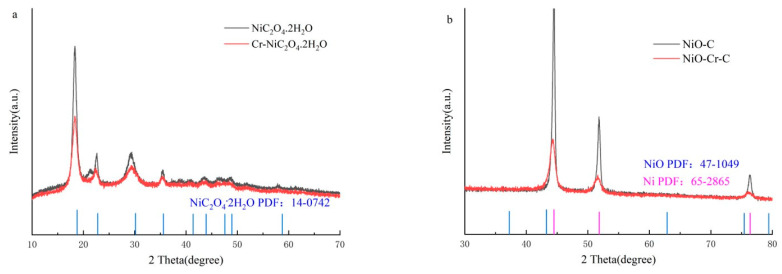
The XRD patterns of the as-prepared composites (**a**) before annealed, (**b**) after annealed.

**Morphological and Structural Characterizations.** The catalysts were synthesized by a two-step process due to their facile operation and low cost. The Cr-NiC_2_O_4_/NF precursor is first obtained by heating homogeneous absolute ethanol and diethyl oxalate solution containing a certain amount of Ni(NO_3_)_2_·6H_2_O and Cr (NO_3_)_3_·9H_2_O at 100 °C; the final NiO-Cr-C/NF can then be formed via thermal annealing treatment of Cr-NiC_2_O_4_/NF precursor at 400 °C in an argon ambiance. Figure 1a shows the XRD pattern of the catalysts before and after annealing at 400 °C. Before annealing, the three peaks at 18.78°, 22.66°, 24.92° and 28.775° correspond to the crystal planes of (−2 0 2), (0 0 2), (−1 1 2) and (−3 1 2) of NiC_2_O_4_·2H_2_O [JCPDS14-0742 (JCPDS25-0581)]. When doping Cr, the peak intensity of 18.78°, 22.66°, 24.92° and 28.775° declined, and the peak positions of 28.775° shifted to the right in comparison with pure NiC_2_O_4_·2H_2_O. In the annealing process, NiC_2_O_4_·2H_2_O would first decompose to NiC_2_O_4_ and H_2_O, and then NiC_2_O_4_ might sequentially decompose to metal Ni and CO_2_. Furthermore, Ni would react with H_2_O, which generates NiO and H_2_ at the decomposition temperature, in addition, some carbon would be retained in the NiO lattice. The total procedure is shown as Formulas (1)–(3). The XRD pattern of annealed catalysts (Figure 1b) shows a strong metallic Ni peak [JCPDS65-2865], and the doping of Cr and C causes a slight shift in the peak position. However, the characteristic peak of NiO [JCPDS47-1049] was too weak and may be covered by the background signal, thus other methods need to be introduced for further analysis.

(1) $${\mathrm{NiC}}_{2}O_{4}\cdot 2H_{2}O\to {\mathrm{NiC}}_{2}O_{4}+2H_{2}O$$

(2) $${\mathrm{NiC}}_{2}O_{4}\to \mathrm{Ni}+2{\mathrm{CO}}_{2}$$

(3) $$\mathrm{Ni}+2H_{2}O\to \mathrm{NiO}+2H_{2}$$

**Figure 2:**
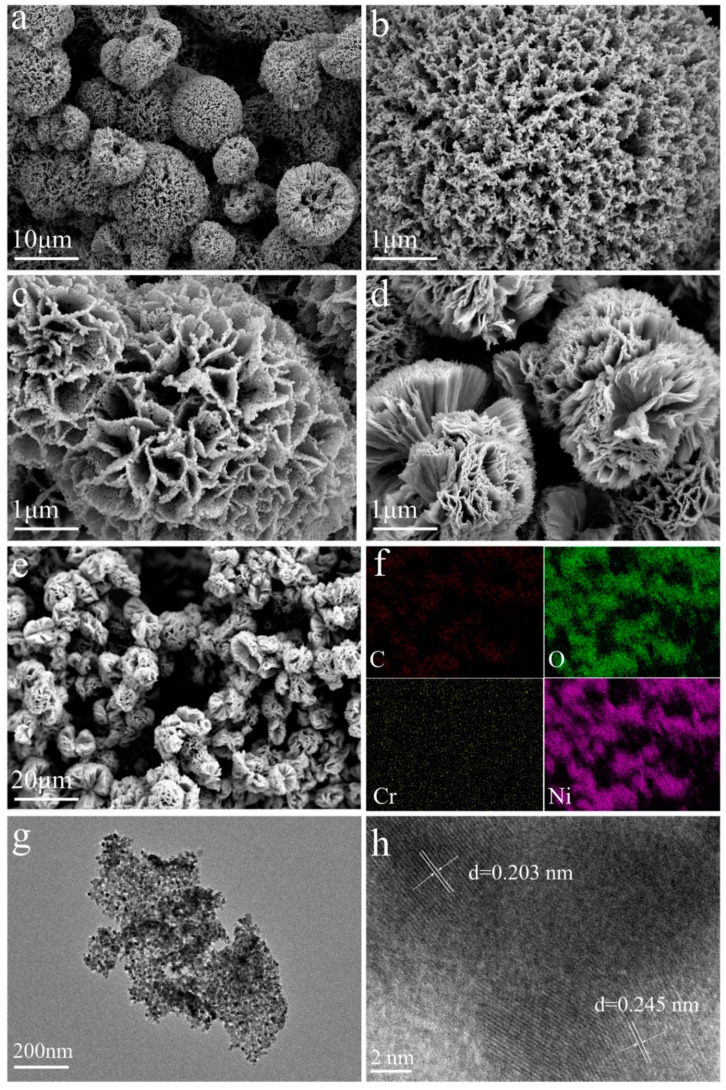
(**a**) SEM image of NiO-Cr-C/NF-3 sample. (**b**–**d**) SEM images of NiO-Cr-C/NF-1, NiO-Cr-C/NF-2 and NiO-Cr-C/NF-3. (**e**,**f**) SEM image and the corresponding elemental mapping of Ni, Cr, O and C. (**g**,**h**) TEM images of NiO-Cr-C/NF-3 in different magnifications.

The morphology and microstructure were also observed by scanning electron microscopy (SEM) and transmission electron microscopy (TEM). After annealing, cellular microspheres with a diameter of about 10 μm are uniformly distributed on the surface of the 3D nickel foam illustrated in Figure 2a, and the microspheres are composed of 2D nanosheets (Figure 2b–d). The 2D → 3D compound structure provides a larger surface area and exposes more active sites, which facilitates the catalytic reaction process. Figure 2b–d show the morphology of the catalysts with different ratio of Ni(NO_3_)_2_·6H_2_O and Cr (NO_3_)_3_·9H_2_O. Porous nanosheets can be observed when no Cr (NO_3_)_3_·9H_2_O was added in the synthetic process (Figure 2b), which may be caused by the formation of CO_2_ while NiC_2_O_4_·2H_2_O decomposed at high temperature. However, the nanosheets turn to be smoother when the mole ratio of Ni(NO_3_)_2_·6H_2_O and Cr (NO_3_)_3_·9H_2_O reaches 7:3 (Figure 2d). In addition, the energy-dispersive X-ray (EDX) pattern exhibits obvious signals of Ni, Cr, C, and O (Figure 2e,f). The EDX elemental mappings in Figure 2f show that Ni, Cr, C, and O elements are distributed uniformly in the catalysts. Figure 2g displays that nanoparticles are uniformly distributed on 2D nanosheets, and these nanoparticles may be assigned to metallic Ni, which is consistent with the results of XRD. As shown in Figure 2h, the interplanar distances of 0.245 nm and 0.203 nm are corresponding to the (1 1 1) plane of NiO and the (1 1 1) plane of Ni. The above results confirm that self-supporting NiO-Cr-C with a 2D → 3D compound structure is successfully fabricated on nickel foam, suggesting the availability and effectiveness of the design strategy in this research. In other words, the novel cellular nanostructure and the potential synergistic effect of Cr and C atoms could be in favor of its electrocatalysis performance.

**Figure 3:**
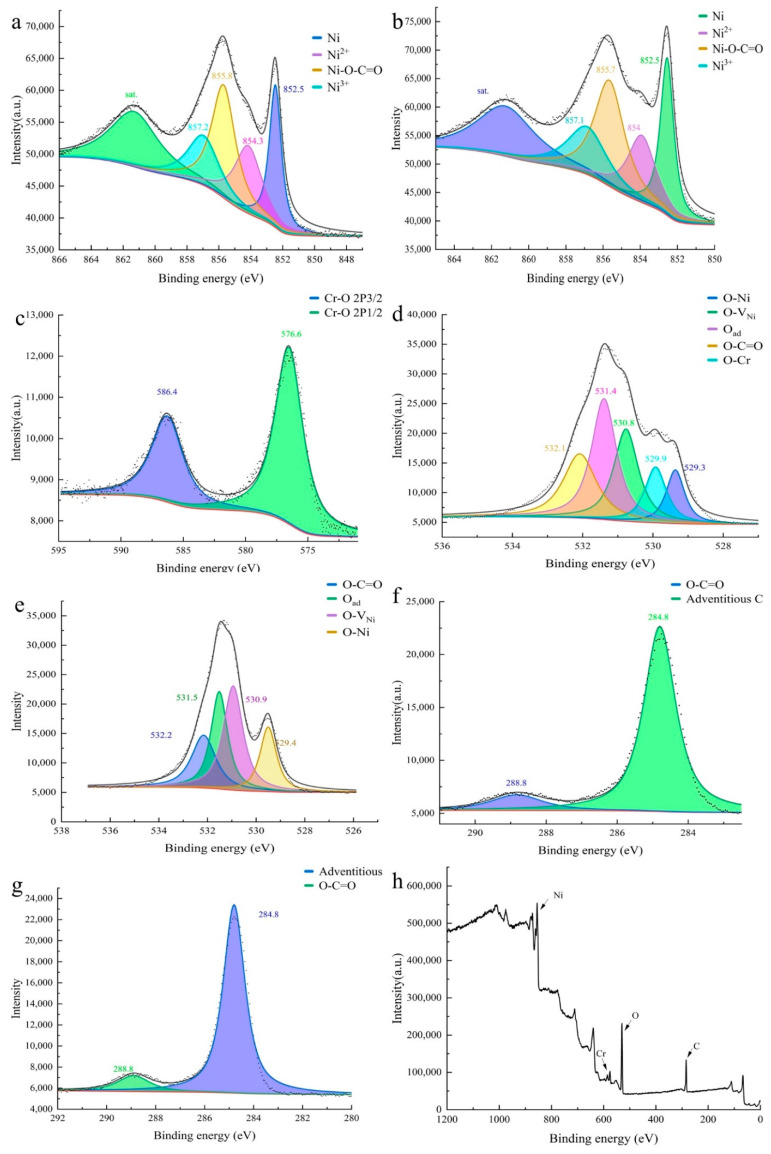
(**a**,**c**,**d**,**f**) High-resolution XPS spectra of the Ni 2p, Cr 2p, O1s and C 1s core levels for NiO-Cr-C/NF-3. (**b**,**e**,**g**) High-resolution XPS spectra of the Ni 2p, O 1s and C 1s core levels for NiO -C/NF. (**h**) XPS survey spectrum of NiO-Cr-C/NF-3.

X-ray photoelectron spectroscopy (XPS) was employed to investigate the surface chemical state, molecular structure and other information of the catalyst. Figure 3h is the XPS survey spectrum, from which the peaks of Ni, O, Cr, and C can be clearly observed. 

In the high-resolution XPS spectrum of Ni 2p (NiO-Cr-C/NF-3, Figure 3a), there are three broad peaks centered at 862 eV (satellite peak), 856 and 852 eV, respectively. The latter two peaks can be further deconvoluted into four sub-peaks. The peak at a binding energy of 852.5 eV is attributed to metallic nickel [33,34,35], and the peak at a binding energy of 854.3 eV is attributed to Ni^2+^ ascribed to NiO [36], while the peak at a binding energy of 857.2 eV is attributed to Ni^3+^, which is due to the Ni vacancy on the surface of NiO [37,38,39,40]. The peak at a binding energy of 855.8 eV is attributed to Ni-O-C=O [21], and the coordination environment of Ni was adjusted by O-C=O groups, which is beneficial to HER as proved by the later DFT simulations. Figure 3b shows the high-resolution XPS spectrum of Ni 2P (NiO-C/NF). The main difference with the spectrum of NiO-Cr-C is the peak position of Ni^2+^ negatively shifted (854.3 eV→854 eV), indicating the doped Cr element reduces the electron cloud density of Ni. The electron of O is more likely biased to Cr^3+^ rather than Ni^2+^, thus lowering the electron cloud density of Ni^2+^. The high-resolution XPS spectrum peaks of Cr 2p (NiO-Cr-C/NF-3, Figure 3c) at 576.6 and 586.4 eV are 2p3/2 and 2p1/2 orbital, respectively, which suggests the existence of Cr^3+^ and Cr-O bonds [41,42,43,44,45]. Usually, the doping of the Cr element increases the electron cloud density of Ni^2+^ due to the electronegativity of Cr being smaller than that of nickel. The reason for this phenomenon probably is that Cr mainly exists in the form of a trivalent state, and the electron adsorption of the O element is strong, which further reduces the electron cloud density of Ni.

Figure 3d is the high-resolution XPS spectrum of O 1s (NiO-Cr-C/NF-3). The deconvoluted peak located at 529.3 eV is attributed to the O-Ni bond [46], which corresponds to the peak at 854.3 eV in the Ni 2p spectrum of Figure 3a. The peak located at 529.9 eV is attributed to the O-Cr bond [33], which is consistent with the Cr 2p spectrum in Figure 3c. Besides, the peak at 530.8 eV should be assigned to O which is adjacent to the Ni vacancy [37]. On account of the existence of Ni^3+^, the Ni vacancy may present to achieve electrical neutrality, which is also mutually confirmed with the Ni^3+^ peak at 857.2 eV in the Ni 2p spectrum in Figure 3a. In addition, the peak at 532.1 eV is indicated as the O-C=O group, which is in agreement with the peak at 288.8 eV in the C 1s spectrum (NiO-Cr-C/NF-3, Figure 3f) [21]. Figure 3e is the high-resolution XPS spectrum of O 1s (NiO-C/NF). The key distinction between Figure 3d and Figure 3e is that there is no O-Cr peak in Figure 3d. Moreover, a conclusion can be drawn from Figure 3d,e that the dopped Cr decreases the binding energy of O 1s orbital by 0.1 eV and increases the binding energy of Ni 2p orbital on the contrary (Figure 3a,b). Thus, the successful doping of the Cr element and the modified electron distribution of NiO-C by Cr could be confirmed ulteriorly.

Lastly, Figure 3f and Figure 3g are the high-resolution XPS spectrum of C1s (NiO-Cr-C/NF-3 and NiO-C/NF), 284.8 eV is due to the adventitious carbon [47,48,49], and the 288.6 eV signal shows the presence of the O-C=O group [21], which is in accordance with spectrum Ni 2p and O 1s.

The XPS results confirmed the existence of the NiO phase, which was not observed in the XRD. The Ni-O-C=O peak in the XPS spectrum indicates that Ni atoms were partly replaced by C atoms on the catalyst surface. The crystal structure near the C atom will be distorted to a certain extent due to the C-O bond length is 1.4 Å, which is smaller than that of the Ni-O bond (2.1 Å); meanwhile, the distribution of electron cloud density of the surrounding Ni atoms will also be greatly affected. The Cr-O bond length is 2.06 Å, which is very close to the Ni-O bond length, so the Cr atom has little effect on the crystal surface structure; the Cr doping makes the Ni^2+^ peak slightly shifted, indicating that Cr mainly plays the role of fine-tuning the electronic structure of surface Ni atoms.

**Electrocatalytic performances of as-prepared materials.** To evaluate the catalytic activity of NiO-Cr-C/NF, Ni foam, Ni foam loaded commercial Pt/C (10 wt% of Pt) catalyst (10% Pt/C/NF) were used as benchmarks. Four kinds of catalysts, named NiO-C/NF, NiO-Cr-C/NF-1, NiO-Cr-C/NF-2 and NiO-Cr-C/NF-3, respectively, with a molar ratio of Ni and Cr of 10:0, 9:1, 8:2 and 7:3, were thoroughly compared in the polarization characteristics. Besides, all the data were acquired without iR drop compensation.

**Figure 4:**
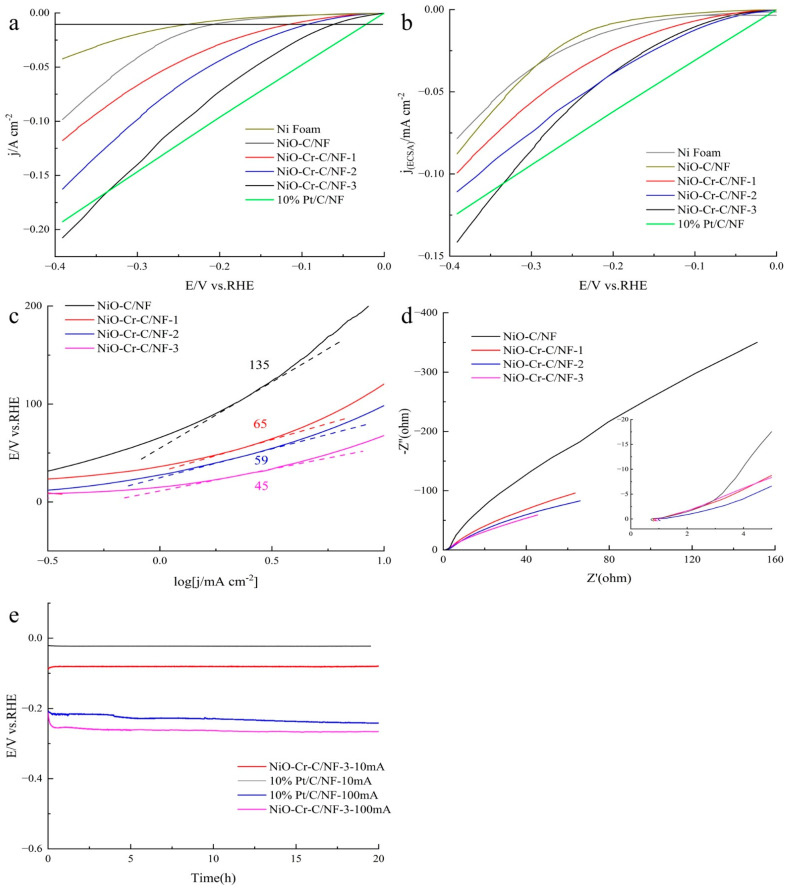
(**a**) HER polarization curves. (**b**) HER polarization curves after ESCA normalized. (**c**) Polarization curve derived Tafel plots (**d**) Electrochemical impedance spectra of prepared samples. (**e**) Chronopotentiometric curve of NiO-Cr-C/NF-3.

Figure 4a is the linear sweep voltammetry (LSV) polarization curves of various samples. The initial overpotential of NiO-Cr-C/NF-3 is rather low, which can be compared with 10% Pt/C/NF. NiO-Cr-C/NF-3 displays the HER activity just behind 10% Pt/C/NF, as manifested by the l overpotential of 69 mV (vs. RHE), the 10% Pt/C/NF of 23 mV (vs. RHE), the Ni foam of 250 mV (vs. RHE) at 10 mA·cm^−2^, which means that the reaction tendency of NiO-Cr-C/NF-3 is lower than 10% Pt/C/NF but higher than the other samples. At the same current density of 10 mA·cm^−2^, the overpotentials of NiO-C/NF, NiO-Cr-C/NF-1 and NiO-Cr-C/NF-2 are 205 mV, 113 mV and 92 mV, respectively. The performance of the CoP-NiO-Cr-C/NF-3 catalyst is inferior to noble metal electrocatalysts [50,51,52,53]; however, it is superior to the most reported NiO-based electrocatalysts [22,54,55,56,57] (Table 1). NiO-Cr-C/NF-3 and 10% Pt/C/NF reach a current density of 208 mA·cm^−2^ and 191 mA·cm^−2^, respectively, when the overpotential is 400 mV. Notably, the electrocatalytic activity of NiO-Cr-C/NF-3 is superior to previously reported transition metal-based electrocatalysts [17,20,22] and even better than 10% Pt/C/NF catalyst at relatively high overpotential. Electrochemical active surface area (ECSA) is an important parameter in exploring the source of catalyst activity. The larger the electrochemical surface active area, the more active sites the catalyst has, and the stronger its catalytic activity will be. We measured the electrochemical double layer capacitance (C_dl_) of the material to estimate its electrochemical surface active area. The ECSA value of Ni foam, 10% Pt/C/NF, NiO-C/NF, NiO-Cr-C/NF-1, NiO-Cr-C/NF-2 and NiO-Cr-C/NF-3, respectively, are 71.2 cm^2^, 1551.0 cm^2^, 540.0 cm^2^, 1184.5 cm^2^, 1150.3 cm^2^, 1873.5 cm^2^. In other words, the NiO-Cr-C/NF-3 catalyst owns the biggest electrochemical active surface area (ECSA) in the four self-prepared samples. To evaluate the intrinsic activity, the current was normalized to ECSA(Figure 4b), which shows that the overpotential of all the samples tends to be converged on a concurrent value at 10 mA·cm^−2^. Moreover, after being normalized to ECSA, NiO-Cr-C/NF-3 and 10% Pt/C/NF reach a current density of 0.14 mA·cm^−2^ and 0.125 mA·cm^−2^, respectively, when the overpotential is 400 mV, which indicates that NiO-Cr-C/NF-3 has higher reaction rate than 10% Pt/C/NF.

The close Tafel slopes could be used to analyze the catalytic reaction kinetics of HER. As shown in Figure 4c, the NiO-Cr-C/NF-3 exhibits a small Tafel slope of 45 mV·dec^−1^, slightly lower than that (135, 65, and 59 mV·dec^−1^) of NiO-C/NF, NiO-Cr-C/NF-1 and NiO-Cr-C/NF-2. The results of the close Tafel slope indicate that NiO-Cr-C/NF-3 has more advantages in reaction kinetics. Moreover, the HER reactions on these catalysts may follow a similar Volmer–Heyrovesky mechanism. In the Volmer step, the O-H bond of H_2_O is broken and adsorbed H atoms (M + H_2_O + e→M-Hads + OH-) are formed on the surface of the electrocatalyst. In the Heyrovsky step, H atoms are adsorbed on the surface and one of the H atoms in H_2_O forms H_2_ (M-Hads + H_2_O + e→M + H_2_ + OH-). Then, the hydrogen generated by the reaction continuously accumulates on the surface of the electrocatalyst, and finally, bubbles are formed to escape the solution. Therefore, synergistic adjustment of the electronic structure of the electrocatalyst to achieve enhanced water adsorption/dissociation and optimized hydrogen adsorption capacity can significantly improve HER activity. Obviously, the Tafel slope of NiO-Cr-C/NF-3 is close to the Heyrovesky step; however, NiO-Cr-C/NF-2, NiO-Cr-C/NF-1 and NiO-C/NF increase sequentially and gradually approach the Volmer step.

In order to evaluate the charge transfer kinetics of the catalysts and explain the electrocatalytic activity, electrochemical impedance spectroscopy was tested and shown in Figure 4d. The charge transfer resistance of the electrocatalysts follows the order: NiO-Cr-C/NF-3 < NiO-Cr-C/NF-2 < NiO-Cr-C/NF-1 < NiO-C/NF. Compared with the non-doped Cr catalyst, the charge transfer resistance of the Cr-doped catalyst has a certain extent reduction, which shows that the Cr doping has a positive significance and effect on the improvement of the catalyst performance. NiO-Cr-C/NF-3 has the lowest charge transfer resistance, which means it has the most favorable charge transfer kinetic conditions. Moreover, in the high frequency range, the linear part has a similar slope, which reveals that the mass transfer characteristics inside the electrode are basically the same.

High stability is also of great significance for the practical application of electrocatalysts; therefore, the stability test of the catalysts was carried out by chronopotentiometry to verify the electrochemical stability of NiO-Cr-C/NF-3 and 10% Pt/C/NF with the current density of 10 mA·cm^−2^ and 100 mA·cm^−2^, respectively. As shown in Figure 4e, the self-supporting NiO-Cr-C/NF-3 electrode remains relatively stable for more than 20 h with continuous HER at the current density of 10 mA·cm^−2^ and 100 mA·cm^−2^, which demonstrates remarkable long-term durability. In addition, the stability of NiO-Cr-C/NF-3 is slightly superior to 10% Pt/C/NF whether at 10 mA·cm^−2^ or at 100 mA·cm^−2^.

In general, NiO-Cr-C/NF series catalysts doped with Cr have better HER performance than NiO-C/NF without Cr doping, while NiO-Cr-C/NF-3 presents the best activity, which is even superior to 10% Pt/C/NF. It means that as the amount of Cr doping increases, the HER activity of NiO-Cr-C/NF series catalysts is higher. However, it is difficult to be doped when the molar ratio of Cr exceeds 30%. The excellent HER performance of NiO-Cr-C/NF-3 under alkaline conditions could be attributed to: (i) The synergy of C and Cr effectively optimizes electron cloud density of Ni, reduces the reaction energy barrier and improves the adsorption behaviors. (ii) The successfully doped C and Cr boost the conductivity of NiO and accelerate electron transfer. (iii) The 2D/3D cellular nano framework greatly expands the electrochemical active surface area (ECSA) of the NiO-Cr-C/NF-3 catalyst, allowing more efficacious sites to be exposed for electrocatalytic reactions.

**Figure 5:**
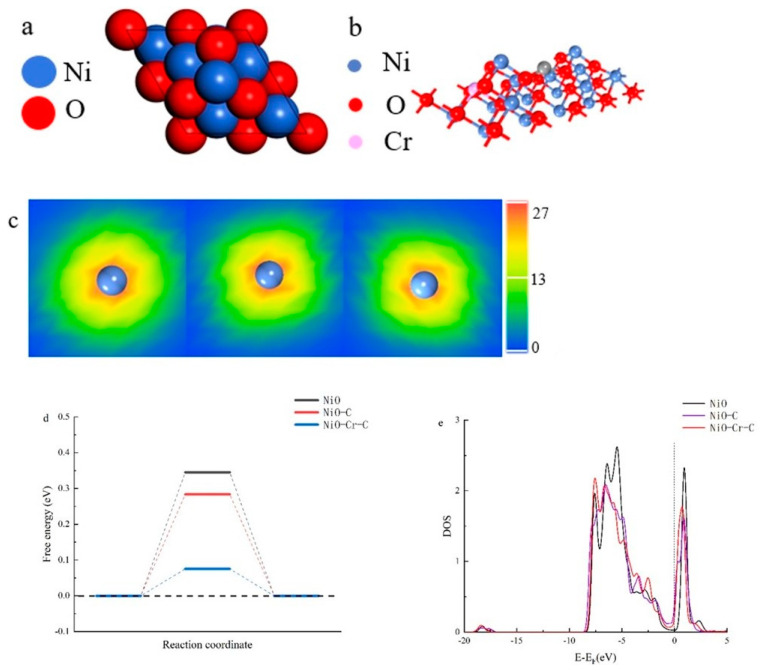
(**a**) Top view of unit cell of Ni terminated NiO (111) surface (Octopolar model). (**b**) Surface structure of NiO-Cr-C. (**c**) Electron cloud density of the top-layer Ni (From left to right represents NiO, NiO-C and NiO-Cr-C). (**d**) Calculated free energy diagram of the HER on NiO, NiO-C and NiO-Cr-C, respectively. (**e**) DOS plots of the top-layer Ni 3d.

**Theoretical simulations.** To explain the fundamental mechanisms responsible for the catalytic performance of NiO-Cr-C/NF-3, the density functional theory (DFT) calculations were carried out by using the CASTEP model of Materia Studio. With the determined structure of the material in hand and previously published literature, the structure model was created first. The (111) facet of NiO was observed according to the HR-TEM image, and there are two possible terminations: Ni or O termination. Literature investigation shows that Ni termination is more stable than O termination and the exposed (111) tends to have surface reconstruction in order to achieve thermodynamically stability [58,59,60,61,62]. In addition, there are three models of NiO (1 1 1) facet now: octopolar, alpha, and vacancy models [63,64,65,66]. The Octopolar model is the most stable structure, in which 3/4 of the first layer of Ni atoms and 1/4 of the second layer of oxygen atoms are holes, as shown in Figure 5a. In the surface reconstruction, more Ni ions are missing than O ions, so Ni vacancies appear on the O surface. Therefore, high valence Ni^3+^ sites are generated to balance the charge. On the other hand, trivalent Ni cations are generated to balance the charge, since more Ni atoms are holes than that of O, which is also confirmed by the XPS results.

In order to in depth understand the electronic structure of Cr and C doped NiO, the projected density of states (PDOS) was calculated as shown in Figure 5. The density of states (DOS) at the Fermi level (E_F_) is higher when C was doped into NiO, which means higher measured conductivity and better charge transfer kinetics. This conclusion is consistent with the EIS data. Besides, DOS at E_F_ of NiO-Cr-C is slightly larger than that of NiO-C, indicating that C doping may play a central role in strengthening the electron transfer kinetics.

The C doping form in the NiO lattice is shown in Figure 5b. Ni is 6-coordinated in NiO, while C doping leads to the distortion of the local structure of NiO, which may be the reason for the mismatch of radius and coordination number between carbon and Ni. This deformation produces sufficient tensile strain on the Ni-O bond and subsequently breaks the bond. Therefore, it reduces the coordination number of Ni from 6 to 3, thereby increasing the charge density of Ni, where the under-coordinated Ni may act as an active h adsorption site in NiO. In addition, the high affinity of carbon with oxygen groups can promote the adsorption or dissociation of water.

The charge density was calculated to study the electron density of the surface Ni atom. As shown in Figure 5c, carbon dopant obviously brings down electron density of top-layer Ni while Cr-doping slightly reduces the electron density of surface Ni atom, which is in conformity with the above experimental results. Moreover, this electron transfer process could effectively activate the surface Ni atoms as reaction sites.

It is well known that the best HER catalysts possess Δ*G* ≈ 0 (that is, the thermoneutral condition where the change in free energy, *G*, is close to zero) so that there is a driving force on the active site; meanwhile, the binding energy should be low enough to facilitate desorption of hydrogen. It can be seen in Figure 5d that the free energy for hydrogen adsorption for NiO-Cr-C is close to thermoneutrality (∆G = 0.11 eV). More importantly, the free energy of adsorbed hydrogen is 0.28 eV (NiO-C) and 0.34 eV (NiO). These results illustrate that hydrogen desorption is easier on the Ni atom of NiO-Cr-C, and the Heyrovsky reaction on the NiO-Cr-C surface is more likely to occur, which achieves the synergistic effect of Ni, Cr and C in the alkaline HER catalysis process.

The DOS plots show that C doping significantly reduces the band gap. This indicates that the conductivity of NiO is improved after doping C, which is favorable for electron transport in HER. Further analysis shows that the narrow band gap is caused by most conduction band minimum falling below the Fermi level and overlapping with the valence band maximum (Figure 5e). The change in DOS can be attributed to the change of the local structure of the top layer Ni mediated by C doping. One of the three Ni-O bonds on the O surface is destroyed, which is due to the strong tension exerted by the nearby short C-O bonds, which gives the top layer Ni of the C surface a higher electron density. The DOS changes in the top layer Ni on the O surface and the C surface also confirmed the influence of C doping, because most of the conduction band minima also moved down below the Fermi level and mixed with the valence band maxima, resulting in their upward movement near the Fermi level. The comparison of the charge density maps of the top layer Ni sites on the O surface and the C surface showed clear evidence that the electron density of the top layer Ni was greatly increased after C doping (Figure 5c).

In general, the DFT calculations prove that the co-doping of Cr and C elements regulate the electronic state distribution structure of the NiO catalyst surface, reduces the Ni d orbital energy level and essentially enhances the interface electronic coupling between NiO and water molecules, further increasing the dissociation kinetics of water. It is consistent with the results of EIS and XPS that trivalent Cr generated by Cr doping is different from the traditional transition metal doping form. In the unique coordination structure, electrons in the surrounding Ni are transferred to Cr, which effectively reduces the d-band energy level of NiO and eventually accelerates the kinetics of water electrolysis. In addition, the exposed Ni sites on the surface can also promote the desorption of H* from the catalyst surface and achieve the synergistic effect of basic HER catalysis.

## 4. Conclusions

In order to improve the HER electrocatalytic activity and stability of non-precious metal catalysts, Cr and C atoms co-doping self-supporting 3D cellular nanospheres which consisting of 2D nanosheets (NiO-Cr-C/NF) were obtained by heating the by hydrothermal fabricated Cr-NiC_2_O_4_/NF precursor at 400 °C. Structural characterizations indicate that the electron of O is more likely biased to Cr^3+^ rather than Ni^2+^, thus lower the electron cloud density of Ni^2+^which further reduces the electron cloud density on the Ni bonded with it. Electrochemical measurements reveal that the co-doping of C and Cr can effectually regulate the distribution structure of the electronic state on the NiO catalyst surface. NiO-Cr-C/NF-3 displays outstanding HER performance in alkaline electrolytes; the optimized catalyst requires an overpotential as low as 69 mV to deliver a current density of 10 mA·cm^−2^. Moreover, at an overpotential of 400 mV, the current density of the NiO-Cr-C/NF-3 catalyst was 0.14 mA∙cm^−2^ compared to 0.125 mA∙cm^−2^ for the 10% Pt/C/NF catalyst after the normalization of ECSA. Moreover, DFT calculations confirm that the synergistic effect primarily resulted from the co-doped Cr and C elements, further revealing the relationship of structure activity between surface electronic configuration and catalytic efficiency. The Cr doping modulates the electron density on the surface of Ni atoms, meanwhile, C doping heightens the conductivity of the catalyst, thereby accelerating the water splitting. Such a hydrothermal-annealing two-step method provides a new avenue to prepare specific 3D self-supporting Cr and C co-doping NiO-Cr-C/NF for the design of new HER catalysts in alkaline media.

## Figures and Tables

**Table 1:** Comparison of electrocatalytic performance for various noble metal electrocatalysts and NiO-based electrocatalysts for HER at 10 mA·cm^−2^.

Catalysts	Electrolyte	*j* (mA∙cm^−2^)	*η* (mV)	References
5-Pt/Ni–P/NF	1 M KOH	10	22	[50]
Ir-Ni/NiO@CNT	1 M KOH	10	24.6	[51]
Er_2_O_3_/Ni-NiO	1 M KOH	10	39	[52]
NiO-Rh2P	1 M KOH	10	46	[53]
NiO/Al_3_Ni_2_	1 M KOH	10	66	[54]
NiO-Cr-C/NF-3	1 M KOH	10	69	This work
CuO-NiO/CN@GP	1 M KOH	10	76.2	[55]
NiOx@BCNTs	1 M KOH	10	79	[22]
NiO nanosheets	1 M KOH	10	83	[56]
Ni/NiO-cp	1 M KOH	10	124	[57]

## Data Availability

The data used in this research are properly cited and reported in the main text.
